# Evaluation of Cardiac Complications Following Hemorrhagic Stroke Using 5-Year Centers for Disease Control and Prevention (CDC) Database

**DOI:** 10.3390/jcm7120519

**Published:** 2018-12-05

**Authors:** Chun-Yu Cheng, Chia-Yu Hsu, Ting-Chung Wang, Ya-Chung Jeng, Wei-Hsun Yang

**Affiliations:** 1 Department of Neurosurgery, Chang Gung Memorial Hospital, Chiayi 61363, Taiwan; chuntoo@gmail.com (C.-Y.C.); northernblotting@gmail.com (T.-C.W.); 2 College of Medicine, Chang Gung University, Taoyuan 33302, Taiwan; mr8898@gmail.com; 3 Department of Neurosurgery, University of Washington, Seattle, WA 98104, USA; 4 Department of Biomedical Sciences and Institute of Molecular Biology, National Chung Cheng University, Chiayi 62102, Taiwan; 5 Department of Neurology, Chang Gung Memorial Hospital, Chiayi 61363, Taiwan; 6 Department of Family Medicine, Taipei Medical University Hospital, Taipei 11031, Taiwan yachungjeng@gmail.com

**Keywords:** cardiac complications, myocardial infarction, arrhythmias, hemorrhagic stroke, death certificates

## Abstract

Literature regarding cardiac deaths in hemorrhagic stroke patients is few. The aim of this study was to investigate the incidence and risk factors of cardiac death in hemorrhagic stroke patients. We used the multiple causes of death database from the Centers for Disease Control and Prevention Wide-ranging Online Data of the United States. We identified death certificates from 2006 to 2010 with hemorrhagic stroke (International Classification of Disease, Tenth Revision (ICD-10) code I60-62), or ischemic stroke (ICD-10 code I63), and evaluated the frequency and risk factors of reporting MI (ICD-10 code I20-25) or arrhythmias (ICD-10 code I44-45, I47-49) as the main cause of death in these populations. Over the five-year period, 224,359 death certificates that mentioned hemorrhagic stroke were identified, and the cause of death was MI in 8.95% and arrhythmia in 7.28% patients. With autopsy confirmation, the incidences of MI and arrhythmias in the hemorrhagic stroke group were still lower than the ischemic group. The odds ratio of reporting arrhythmias as a cause of death in hospitalized population was higher. A substantial percentage of hemorrhagic stroke patients had cardiac death. Greater efforts are needed to closely monitor high-risk groups such as females and the elderly.

## 1. Introduction

Serious cardiovascular events such as myocardial infarction (MI), atrial fibrillation and ventricular arrhythmias (VA) are common complications in the acute period after a stroke and may increase the risk of sudden death [1,2,3]. The increased incidence of cardiac arrhythmias in 50% of patients following an acute stroke has been identified by electrocardiographic (ECG) alternation [4]. Patients with a higher cardiac risk require more aggressive treatment to improve survival rates and reduce morbidity [5]. As a result, cardiac monitoring to identify changes in cardiac rhythm before symptoms occur is quite important and a greater emphasis should be placed on stroke guidelines approved by the American Stroke Association [6]. VA may be triggered by cerebrogenic autonomic dysfunction and neuro-hormone imbalance after a stroke by a direct influence on the central nervous system, with synergistic catecholamine surge leading to myocardial infarction and arrhythmias [7,8]. Recent studies have also shown that the insula cortex plays a critical role in triggering autonomic dysregulation and the development of cardiac complications in the acute stage following a stroke [9,10]. However, it is difficult to identify if mortality is caused by cardiac complications or stroke in patients following a hemorrhagic stroke, especially when reporting of arrhythmias as a cause of death.

There is ample research regarding cardiovascular complications in ischemic stroke, but only a few have explored the cardiovascular complications following a hemorrhagic stroke [4,10,11,12,13,14,15]. Hemorrhagic stroke constitutes approximately 10%–20% of all strokes [16] with a high mortality rate up to 40% [17,18,19]. When compared with ischemic stroke patients, hemorrhagic patients are younger, have less cardiovascular risk factors, and rarely have a history of cardiovascular disease. Therefore, clinicians might have paid less attention to cardiac complications in these patients. In addition, the use of antiplatelets and anticoagulants to treat cardiac complications in the acute stage after a hemorrhagic stroke is a controversial issue. Therefore, a better knowledge of cardiac complications following a hemorrhagic stroke may help physicians to closely monitor and develop preventive strategies in high-risk groups.

In this study, in order to assess the incidence and risk factors of cardiac mortality in hemorrhagic stroke patients, compared to ischemic stroke patients, we attempted to use death certificates database to evaluate the frequency of MI or arrhythmias as a cause of death in patients in the United States from years 2006 to 2010.

## 2. Materials and Methods and Statistical Analysis

We used the multiple causes of death database that was provided by the Centers for Disease Control and Prevention Wide-ranging Online Data for Epidemiological Research (CDC WONDER). The CDC WONDER database is an easy-to-use system and contains mortality causes based on death certificates with or without autopsy data in all United States counties [20]. The death certificates obtained from the registration data included: (1) The immediate cause of mortality (the main disease directly contributes to the death), (2) up to 20 contributing causes of death, and (3) a single underlying cause of death (the disease or injury that initiated the events resulting in death) and associated demographic data. All information on death certificates is recorded by the physician and coded in accordance with the International Classification of Disease, Tenth Revision (ICD-10). The database should be utilized for health statistical reporting and analysis only. Our study data were all gathered from the database covering the data of period from 2006 to 2010. To perform subgroups analysis, the data were extracted by grouping variables: Calendar year, sex, age, race, and place of death.

In this retrospective study, we extracted patients from the CDC WONDER database according to two criteria: (1) MI (ICD-10 code I20-25) or arrhythmia (ICD-10 code I44-45, I47-49) being the immediate cause of death, (2) with mention of hemorrhagic stroke (ICD-10 code I60-62) or ischemic stroke (ICD-10 code I63) as the contributing cause of death. Because this database does not contain any individual identifiers, appropriate statistical analysis is mostly carried out using aggregate data analysis methods. We then calculated the odds ratio (OR) and 95% confidence interval (CI) by the subgroups categorized by year, age, sex, race and place of death using simple logistic regression with subgroup sample sizes as weighs. To further explore the reliability of the data, we also performed the same analysis in patients reporting MI as the main cause of death with autopsy confirmation. All the data analysis was performed using SAS 9.1.3. Throughout the article, a hypothesis test *p*-value of less than 0.05 was considered statistically significant.

## 3. Results

Between 2006 and 2010, a total of 224,359 death certificates were identified as mentioning hemorrhagic stroke in the United States. Among them, 20,071 (8.95%) reported MI as a cause of death (Table 1). According to demographic data, females vs. males (10.58% vs. 7.48%) and the elderly (those above 65 years vs. those below 65 years, 10.69% vs. 5.31%) had higher odds ratios of reporting MI as the cause of death. When considering the place of death, hospitalized patients had a lower odds ratio (OR: 0.59, 95% CI: 0.56–0.62).

During the same five-year period, 62,971 death certificates were identified as noting ischemic stroke (Table 2). Among them, 13,896 (22.07%) reported MI as the cause of death. According to the demographic data, females vs. males (24.61% vs. 20.14%) and the elderly (those above 65 years vs. below 65 years, 23.46% vs. 15.89%) both had higher odds ratios as well in the hemorrhagic stroke group. In addition, the hospitalized population and those who stayed in nursing homes both had lower odds ratios than those who lived in their homes (OR: 0.6, 95% CI: 0.56–0.63 and OR: 0.68, 95% CI: 0.63–0.72). The difference between races was found not to be significant.

When we compared the data to the autopsy results, the total number of patients decreased (Table 1 and Table 2), but the percentage of MI as a cause of death increased to 12.01% in the hemorrhagic stroke group and 28.52% in the ischemic stroke group. Both in the hemorrhagic and ischemic stroke groups, females and the elderly still had higher odds ratios, but the risk in hospitalized population showed a decrease.

When arrhythmia was noted as the cause of death, females and the elderly had higher odds ratios in hemorrhagic and ischemic stroke groups (Table 3). In addition, hospitalized population and patients living in nursing homes had higher odds ratios to die as arrhythmias in the hemorrhagic stroke group (OR: 1.24, 95% CI: 1.14–1.33 and OR: 1.81, 95% CI: 1.66–1.98). In the ischemic stroke group, hospitalized population had the highest odds ratio (OR: 1.15, 95% CI: 1.07–1.23).

## 4. Discussion

This is a unique study that attempted to estimate the incidence of cardiac complications in patients with hemorrhagic stroke. In this study, we identified 224,359 death certificates that mentioned hemorrhagic stroke. Among them, 20,071 (8.95%) were reported as dying from MI and 16,330 (7.28%) from cardiac arrhythmia. During the same five-year period, we also identified 62,971 death certificates that mentioned ischemic stroke. Among these patients, 13,896 (22.07%) were reported as dying from MI and 11,378 (18.07%) as dying from cardiac arrhythmia. At the same time, we found that being female or being elderly were risk factors for developing cardiac complications in both the hemorrhagic and ischemic stroke groups. In our study, the incidence of MI was analyzed according to death certificates that were prescribed by physicians. From the analysis, the incidence of MI in the hemorrhagic stroke group was lower than that in the ischemic stroke group. However, because of the larger population in the hemorrhagic stroke group (224,359 vs. 62,971), the number of patients reporting MI as the cause of death is higher in the hemorrhagic stroke group than the number in the ischemic stroke group (20,071 vs. 13,896). Thus, the large number of deaths that resulted from MI is worth noting.

The lower number in the ischemic stroke group is possibly the result of advances in anticoagulant medication and neuro-interventional treatment in recent decades; furthermore, a greater number of people had better awareness of the early signs of strokes such as transient ischemic stroke or lacunar stroke. On the contrary, the treatment of hemorrhagic stroke has not seen such dramatic advances and the mainstay of treatment is still the control of blood pressure and aggressive management of raised intracranial pressure. Not only is there a lack of effective treatment at this time, the hemorrhagic stroke itself usually causes an immediate and disastrous outcome. Most of the hemorrhagic patients have disabilities even after aggressive treatment.

This high risk of developing MI in ischemic stroke patients is well documented [21]. Pasternak et al. has reported that ischemic stroke and MI both have several common risk factors [22]. When compared with the ischemic stroke group, the incidence of reporting MI as the cause of death is lower in the hemorrhagic stroke group, according to our study results. Although we identified similar risk factors including gender and age in both groups, there must be other factors existing between hemorrhagic stroke and MI. Another possible explanation that may account for this discrepancy between the hemorrhagic and ischemic stroke groups is that hemorrhagic stroke cases enrolled in our analysis could be caused by a variety of cerebrovascular pathologies. Not only hypertensive induced intracerebral hemorrhage, aneurysm, arteriovenous malformation and tumors can also cause acute hemorrhagic stroke, and these differences could not be identified based on the death certificates alone and as such becomes a source of potential bias. We believed that patients with hemorrhagic stroke caused by non-hypertensive pathology may have more cardiac complications [23,24,25].

Another cardiac related complication is arrhythmias. Associations between cardiac arrhythmias and ischemic stroke have also been reported in many studies [26,27,28,29]. Lavy et al. reported a 39% incidence of new onset cardiac arrhythmias after stroke [13]. However, studies have seldom reported on the relationship between arrhythmias and hemorrhagic stroke. Yamour et al. reported new onset ventricular arrhythmia in 10% of patients with intracerebral hemorrhage [15]. In subarachnoid hemorrhagic patients, the incidence of cardiac arrhythmia could be as high as 98% [30]. Surprisingly, cardiac-associated mortalities have been reported as high as 80% in cases of acute stroke [31]. In our analysis, the incidence of dying from arrhythmia among patients who died with stroke was 7.28% in the hemorrhagic stroke group and 18.7% in the ischemic stroke group. Similar to MI, the risk is higher among females and the elderly population. Engström et al. also reported that ventricular arrhythmias are common among older individuals, in even those without atherosclerotic disease [32]. In our study, patients with cerebral infarction seems to have an additional risk of developing arrhythmias, and this finding is consistent with other studies that identified an association between right-sided hemispheric infarction and cardiovascular function derangement [7,33,34,35].

We also compared our analysis to autopsy-confirmed cases. There were no significant changes in the results when evaluating the incidence of MI as a cause of death on death certificates with mention of stroke. Similarly, females and the elder population still demonstrated a higher risk group. However, when we tried to compare the arrhythmia cases with autopsy records, the case number became less than 300 in the five-year period examined and this made further analysis rather unreliable. There are two possible explanations for this dramatic drop in case number. First, it is difficult to confirm arrhythmias as a cause of death with autopsy records. Second, not every clinical physician may think it is necessary to perform autopsy to confirm the real cause of death.

The strengths of this study include the generalizability of the population-based study design regarding all patients in different places of death with stroke during the study years. Besides, we could also infer that there is a higher possibility of cardiac complications among female and elderly group with stroke as a cause of death. Using the autopsy-confirmed data to evaluate the death of cause also enhances the reliability of our analysis. In this study, we conducted an analysis based on the information reported in death certificates. However, there are a number of limitations that should be noted. First, the interval of cardiac death following hemorrhagic stroke could not be confirmed from the CDC WONDER database due to the lack of individual identifiers. MI or arrhythmia could have occurred several months after the attack of ischemic or hemorrhagic stroke. Therefore, the clinical physicians would not report stroke on the death certification and this could result in the underestimation of the stroke population. Although we tried to retrieve the death certificates during the same hospitalization, the incidence of MI or arrhythmia might be overestimated in our analysis. Second, defining the cause of death in stroke patients is difficult especially when there is a large variation between experts in determining whether the death is stroke related [36,37,38]. Besides, the risk factors of cardiac death, such as hypertension, hyperlipidemia and congestive heart failure, are not included in our study and physicians may not address these diseases in the death certificates if these risk factors are not directly related to the cardiac death. Therefore, these risk factors are easily underestimated by using the death database. The mechanism of these risk factors leading to cardiac death in hemorrhagic stroke patients is out of scale of this study and needs further investigation. Despite that, the purpose of our study is to focus on the prevalence of acute cardiac events following hemorrhagic stroke. Third, there is unavoidable selection bias while using death certificate data. This kind of bias has been called Berksonian bias and arises when both outcome and exposure status could affect the enrolled population [39]. This selection bias could cause an overestimation between the two conditions, for instance, cerebrovascular disease and cardiovascular complications [40]. Because of the limitations mentioned above, caution should be taken in the interpretation of these data and further evaluation is necessary.

## 5. Conclusions

In conclusion, further prospective studies are needed to explore the relationships between cardiac complications and hemorrhagic stroke. By utilizing this database, we identified the incidence of death from MI and cardiac arrhythmias among patients who died with hemorrhagic stroke as 8.95% and 7.28%, respectively. Female and elderly patients tend to develop more cardiac complications following hemorrhagic stroke. Identification of arrhythmias with close cardiac monitoring and its aggressive management would prevent and reduce cardiac catastrophe in patients hospitalized with hemorrhagic stroke.

## Figures and Tables

**Table 1:** The incidence and odds ratio of reporting myocardial infarction (MI) as a cause of death on death certificates with mention of hemorrhagic stroke.

Total		With Mention of Hemorrhagic Stroke	MI as Cause of Death				With Mention of Hemorrhagic Stroke	With Autopsy Confirmed			
			*n*	%	Odds Ratio	95% CI		*n*	%	Odds Ratio	95% CI
Total		224,359	20,071	8.95			14,717	1767	12.01		
Year	2006	44,700	4107	9.19	1.04	0.99–1.08	3154	344	10.91	0.85	0.72–0.99
	2007	44,918	4084	9.09	1.02	0.98–1.07	3162	363	11.48	0.89	0.76–1.04
	2008	44,825	4022	8.97	1.00	0.96–1.06	2835	346	12.20	0.95	0.81–1.12
	2009	44,906	3851	8.58	0.96	0.92–1.01	2812	364	12.94	1.02	0.87–1.20
	2010	45,010	4007	8.90	1.00		2754	350	12.71	1.00	
Sex	Male	118,335	8849	7.48	0.68	0.66–0.70	6388	608	9.52	0.65	0.59–0.72
	Female	106,024	11,222	10.58	1.00		8329	1159	13.92	1.00	
Age	<65 years	72,916	3874	5.31	0.47	0.45–0.49	11,405	1062	9.31	0.38	0.34–0.42
	≥65 years	151,431	16,193	10.69	1.00		3306	702	21.23	1.00	
Race	American Indian or Alaska Native	1284	97	7.55	0.77	0.63–0.95	122	9	7.38	0.53	0.27–1.06
	Asian or Pacific Islander	9229	663	7.18	0.73	0.67–0.79	609	82	13.46	0.82	1.33
	Black or African American	31,982	1897	5.93	0.60	0.57–0.63	3665	338	9.22	0.60	0.77
	White	181,864	17,414	9.58	1.00		10,321	1338	12.96	1.00	
Place of death	Hospitalized	166,238	13,429	8.08	0.59	0.56–0.62	6033	634	10.51	0.70	0.63–0.79
	Nursing home	18,480	2378	12.87	0.99	0.92–1.05	133	26	19.55	1.46	0.94–2.25
	Home	12,835	1673	13.03	1.00		5058	723	14.29	1.00	

CI, confidence interval.

**Table 2:** The incidence and odds ratio of reporting myocardial infarction (MI) as a cause of death on death certificates with mention of ischemic stroke.

Total		With Mention of Ischemic Stroke	MI as Cause of Death				With Mention of Ischemic Stroke	With Autopsy Confirmed			
			*n*	%	Odds Ratio	95% CI		*n*	%	Odds Ratio	95% CI
Total		62,971	13,896	22.07			3177	906	28.52		
Year	2006	13,707	3067	22.38	1.07	1.01–1.13	707	209	29.56	0.97	0.76–1.23
	2007	12,859	2885	22.44	1.07	1.01–1.14	628	174	27.71	0.88	0.69–1.13
	2008	12,375	2709	21.89	1.04	0.98–1.10	622	172	27.65	0.88	0.69–1.13
	2009	12,111	2702	22.31	1.06	1.00–1.13	605	165	27.27	0.86	0.67–1.11
	2010	11,919	2533	21.25	1.00		615	186	30.24	1.00	
Sex	Male	35,869	7225	20.14	0.77	0.74–0.80	1911	269	14.08	0.45	0.38–0.54
	Female	27,102	6671	24.61	1.00		1266	337	26.62	1.00	
Age	<65 years	11,562	1837	15.89	0.62	0.58–0.65	1951	412	21.12	0.40	0.34–0.46
	≥65 years	51,407	12,059	23.46	1.00		1226	494	40.29	1.00	
Race	American Indian or Alaska Native	273	55	20.15	0.87	0.65–1.17	15	2	13.33	0.37	0.08–1.65
	Asian or Pacific Islander	1472	321	21.81	0.96	0.85–1.09	89	30	33.71	1.23	0.79–1.93
	Black or African American	7303	1421	19.46	0.84	0.79–0.89	749	194	25.90	0.85	0.70–1.02
	White	53,923	12,099	22.44	1.00		2324	680	29.26	1.00	
Place of death	Hospitalized	34,468	6850	19.87	0.60	0.56–0.63	1838	441	23.99	0.65	0.54–0.79
	Nursing home	13,998	3071	21.94	0.68	0.63–0.72	141	62	43.97	1.63	1.12–2.35
	Home	7492	2200	29.36	1.00		685	223	32.55	1.00	

CI, confidence interval.

**Table 3:** The incidence and odds ratio of reporting arrhythmia as a cause of death on death certificates with mention of hemorrhagic and ischemic stroke.

Total		With Mention of Hemorrhagic Stroke	Arrhythmia as Cause of Death				With Mention of Ischemic Stroke	Arrhythmia as Cause of Death			
			*n*	%	Odds Ratio	95% CI		*n*	%	Odds Ratio	95% CI
Total		224,359	16,330	7.28			62,971	11,378	18.07		
Year	2006	44,700	3020	6.76	0.85	0.80–0.89	13,707	2260	16.49	0.83	0.77–0.88
	2007	44,918	3091	6.88	0.86	0.82–0.91	12,859	2235	17.38	0.88	0.83–0.94
	2008	44,825	3281	7.31	0.92	0.88–0.97	12,375	2230	18.02	0.92	0.86–0.98
	2009	44,906	3389	7.55	0.95	0.91–1.00	12,111	2354	19.44	1.01	0.95–1.08
	2010	45,010	3549	7.88	1.00		11,919	2299	19.29	1.00	
Sex	Male	118,335	7813	6.60	0.81	0.78–0.84	35,869	4200	11.71	0.37	0.35–0.38
	Female	106,024	8517	8.03	1.00		27,102	7178	26.49	1.00	
Age	<65 years	72,916	1393	1.91	0.18	0.17–0.19	11,562	632	5.47	0.22	0.20–0.24
	≥65 years	151,431	14,937	9.86	1.00		51,407	10,746	20.9	1.00	
Race	American Indian or Alaska Native	1284	37	2.88	0.34	0.24–0.47	273	36	13.19	0.63	0.45–0.90
	Asian or Pacific Islander	9229	599	6.49	0.79	0.72–0.86	1472	273	18.55	0.95	0.83–1.08
	Black or African American	31,982	951	2.97	0.35	0.32–0.37	7303	636	8.71	0.40	0.37–0.43
	White	181,864	14,743	8.11	1.00		53,923	10,433	19.35	1.00	
Place of death	Hospitalized	166,238	11,820	7.11	1.24	1.14–1.33	34,468	6443	18.69	1.15	1.07–1.23
	Nursing home	18,480	1864	10.09	1.81	1.66–1.98	13,998	2334	16.67	1.00	0.93–1.08
	Home	12,835	749	5.84	1.00		7492	1250	16.68	1.00	

CI, confidence interval.

## Abbreviations

(ICH)	Intracerebral hemorrhage
(MI)	Myocardial infarction
(CDC WONDER)	Centers for Disease Control and Prevention Wide-ranging Online Data for Epidemiological Research
(ICD)	International Classification of Diseases
(CI).	Confidence interval
